# Serum inhibin B for differentiating between congenital hypogonadotropic hypogonadism and constitutional delay of growth and puberty: a systematic review and meta-analysis

**DOI:** 10.1007/s12020-020-02582-0

**Published:** 2021-01-19

**Authors:** Yuting Gao, Qin Du, Liyi Liu, Zhihong Liao

**Affiliations:** Department of Endocrinology and Metabolism, Hospital of Sun Yat-sen University, Guangzhou, Guangdong PR China

**Keywords:** Inhibin B, Congenital hypogonadotropic hypogonadism, Constitutional delay of growth and puberty, Meta-analysis

## Abstract

**Purpose:**

The distinction between congenital hypogonadotropic hypogonadism (CHH) and constitutional delay of growth and puberty (CDGP) in patients with delayed puberty is difficult to distinguish, but important for timely treatment. The aim of this study is to perform a systematic review and meta-analysis to determine the diagnostic performance of serum inhibin B (INHB) levels for differentiating CHH and CDGP.

**Methods:**

PubMed, EMBASE, and Cochrane Library databases were systematically searched from the date of database inception to November 10, 2019 for studies examining the use of serum INHB to discriminate between CHH and CDGP. Pooled odds ratios (OR), sensitivity, specificity, and 95% confidence intervals (CI) were calculated. The Quality Assessment of Diagnostic Studies-2 (QUADAS-2) was used to assess the quality of the included studies. Sub-analyses were performed including that based on testicular volume (TV) and study design.

**Results:**

Seven studies, comprising of 349 patients (96 CHH and 253 CDGP), were included in the meta-analysis. For differentiating between CHH and CDGP, INHB level exhibited good diagnostic accuracy with a pooled sensitivity of 92% (95% confidence interval [CI]: 0.86–0.96, *I*^2^ = 0.4%, *p* = 0.4343), specificity of 92% (95% CI: 0.88–0.94, *I*^2^ = 68.1%, *p* = 0.0009), and pooled area under the receiver operating characteristic curve (AUC) of 0.9619. The cut-off values of INHB for boys were 56, 66, 80, 96, 94.7, 111, and 113 pg/ml (assay method standardized to Gen II ELISA). Sub-analyses showed that testicular volume and study design could be a source of statistically significant heterogeneity in specificity. In boys with a testicular volume of ≤3 ml, INHB performed well with a sensitivity of 92%, specificity of 98%, and AUC of 0.9956.

**Conclusion:**

INHB exhibits excellent diagnostic efficiency in distinguishing CHH from CDGP, especially in boys with severe puberty deficiency (TV ≤ 3 ml).

## Introduction

Delayed puberty (DP) is defined as pubertal onset occurring at an age of 2 or 2.5 standard deviations later than the mean of the population. Classically, it refers to the absence of testicular enlargement (volume <4 ml) in boys by age 14 and absent breast development in girls by age 13 [1]. The causes of delayed puberty can be classified into five categories: constitutional delay of growth and puberty (CDGP) (53%), functional hypogonadotrophic hypogonadism (19%), hypogonadotrophic hypogonadism (HH) (12%), hypergonadotrophic hypogonadism (13%), and unclassified (3%) [2]. CDGP is the most common reason for delayed puberty, and is a physiological variant of normal puberty characterized by a slowing of growth and delayed timing of pubertal development [3]. Congenital hypogonadotropic hypogonadism (CHH) is a form of HH that occurs in ~1 in 4000 births. It is a relatively rare heterogeneous disorder caused by deficient production, secretion, or action of gonadotropin-releasing hormone (GnRH). Idiopathic hypogonadotropic hypogonadism (IHH), isolated hypogonadotropic hypogonadism, and isolated GnRH deficiency are similar to CHH, and the terms are often are used interchangeably [4]. The term CHH is commonly used because of the disorders hereditary characteristics. CHH and CDGP share several similar signs and symptoms. Differentiating between the two during early adolescence is challenging, especially in normosmic pre-pubertal boys presenting with pubertal delay without cryptorchidism, because patients with partial CHH present with different degrees of pubertal underdevelopment.

The presence of progressive pubertal development by age 18 is the “gold standard” for differentiating CDGP from CHH. However, an early diagnosis of CHH or CDGP is important because a delayed diagnosis may be harmful to psychological well-being and quality of life, and because of the impact on bone mineralization and fertility [5]. A variety of methods for differentiating the two disorders have been proposed, including nocturnal luteinizing hormone (LH) sampling, testosterone response to human chorionic gonadotropin (HCG), a GnRH-stimulated LH response, measurement of urinary gonadotrophins, and daily urine excretion of follicle-stimulating hormone (FSH) and LH [6–10]. Most of these tests are invasive, time-consuming, imprecise, and/or costly. Likewise, it is hard to standardize the various GnRH, GnRH agonists (GnRHa), and HCG stimulation tests worldwide. The medication doses, injection frequency, and timing of blood sampling also vary in the stimulation tests [11–13], which also makes the results difficult to compare. No single test has emerged that is reliable, easy to perform, and has acceptable sensitivity and specificity for distinguishing between CHH and CDGP.

Serum inhibin B (INHB) level is used for distinguishing CHH from CDGP. Using INHB level avoids the need for repeated injections, and its assay method is relatively simple. INHB is produced in the Sertoli cells of the testis in males, and in the granulosa cells of the ovary in females. It belongs to the transforming growth factor-β (TGF-β) super family, and regulates the synthesis and secretion of FSH in a negative feedback loop [14]. INHB level reflects Sertoli cell number and function [15, 16]. In males, INHB peaks shortly after birth, decreases during childhood, and then increases at puberty due to FSH stimulation. It is a better marker of fertility status than FSH and LH [17]. In females, INHB level is related to the number of antral follicles, and reflects the ovarian response to gonadotrophins [15, 16]. INHB may be used as a regular screening test for certain ovarian cancers (mucinous carcinomas, granulosa cell tumors), especially in post-menopausal women [18].

A number of studies have examined the diagnostic value of serum INHB level for differentiating between CHH and CDGP. However, the sensitivity, specificity, and cut-off values reported in studies are inconsistent. Thus, the purpose of this study was to perform a meta-analysis to determine the diagnostic performance of serum INHB level for differentiating CHH and CDGP.

## Methods and materials

The meta-analysis complied with the Preferred Reporting Items for Systematic Reviews and Meta-Analyses (PRISMA) statement [19].

### Data sources and search strategy

PubMed, EMBASE, and Cochrane Controlled Register databases were searched from inception to November 10, 2019 for studies examining the use of INHB level for differentiating CHH and CDGP. Mesh terms and free-text words were matched and used together for database searches using combinations of keywords as follows: “constitutional delay of growth and puberty [MESH] OR constitutional delay OR constitutional growth delay OR delayed puberty OR CDGP OR CDG OR CDP” AND “congenital hypogonadotropic hypogonadism [MESH] OR hypogonadotropic hypogonadism OR isolated GnRH deficiency OR IHH OR CHH OR HH”. The reference lists of all relevant articles were also searched to identify additional potentially relevant studies. Two reviewers (GYT and DQ) performed the database searches, and disagreements were resolved through discussion or by consulting a third researcher (LZH). If full text of a study could not be found, we contacted the author or the development agency.

### Inclusion and exclusion criteria

Studies were included if they meet all of the following criteria: (1) Published in English; (2) The study examined the use of serum INHB level for differentiating between CHH and CDGP; and (3) Studies reported data as true positive (TP), false positive (FP), true negative (TN), and false negative (FN) rates, or reported overall sample size and sensitivity and specificity values which could be used for statistical analysis.

The exclusion criteria were (1) Animal and in vitro studies; (2) Reviews, meta-analysis, and case reports; (3) Studies that reported unrelated statistical and clinical data; (4) Duplicate articles that were updated versions; (5) The purpose of the study was not related to the objectives of this study; and (6) Studies including patients with functional hypothalamus-pituitary-gonadal (HPG) axis impairment.

### Screening and data extraction

All included studies were imported into ENDNOTE. Studies were first screened by titles and abstracts, and then the full text was assessed using the inclusion and exclusion criteria. The following data were extracted from studies that met the inclusion criteria: name of the first author, publication year, country of publication, journal name, study design, age of participants, whether testosterone priming treatment was performed, testicular volume (TV), INHB measurement method, CHH diagnostic standard, CDGP diagnostic standard, cut-off values, number of CHH patients, number of CDGP patients, and TP, FP, TN, and FN. If data were incomplete for the purposes of the meta-analysis, we contacted the author by mail and asked them to provide the necessary information. Extracted data were recorded in Excel. Screening and data extraction were performed independently by two of the authors (GYT and DQ), and disagreements were resolved through discussion or by consulting a third researcher (LZH).

### Quality assessment

The quality of the included studies was assessed using the Quality Assessment of Diagnostic Accuracy Studies-2 (QUADAS-2) tool [20]. The tool has four main components: patient selection, index testing, reference standard, and flow and timing. Each component was evaluated for risk of bias, and the first three components were also evaluated for applicability. The QUADAS-2 also provides relevant questions to help rate studies in the above-mentioned domains. Quality assessment was independently done by two researchers (GYT and LLY), and disagreements were resolved through discussion or by consultation with a third researcher (LZH).

### Statistical analysis

Meta-Disc version 1.4 (Ramony Cajal Hospital, Madrid, Spain) [21] was used for data analysis. The pooled sensitivity, specificity, diagnostic odds ratio (DOR), and summary receiver operator characteristics (SROC) for INHB in distinguishing the two conditions were calculated. Sensitivity, specificity, and DOR were presented with a 95% confidence interval (CI), and for the SROC area under the curve the standard error (SE) was calculated. The Q* index, which is the point closest to the ideal top-left corner of the SROC space, is defined as exhibiting the best combination of sensitivity and specificity [22]. The inconsistency index (*I*^2^) was used to evaluate heterogeneity among the studies: an *I*^2^ < 25% indicates low heterogeneity, a value of 25–50% indicates moderate heterogeneity and an *I*^2^ > 50% indicates high heterogeneity [23]. When significant heterogeneity was identified (*I*^2^ > 50%) a random-effects model of analysis was used, whereas a fixed-effects model of analysis was used when heterogeneity was low or moderate. The Spearman correlation coefficient was calculated to determine if a threshold effect existed. Meta-regression analysis was then performed to explore the possible sources of heterogeneity in the studies. Statistical significance was defined as a two-tailed value of *p* < 0.05.

## Results

### Literature search

**Fig. 1:**
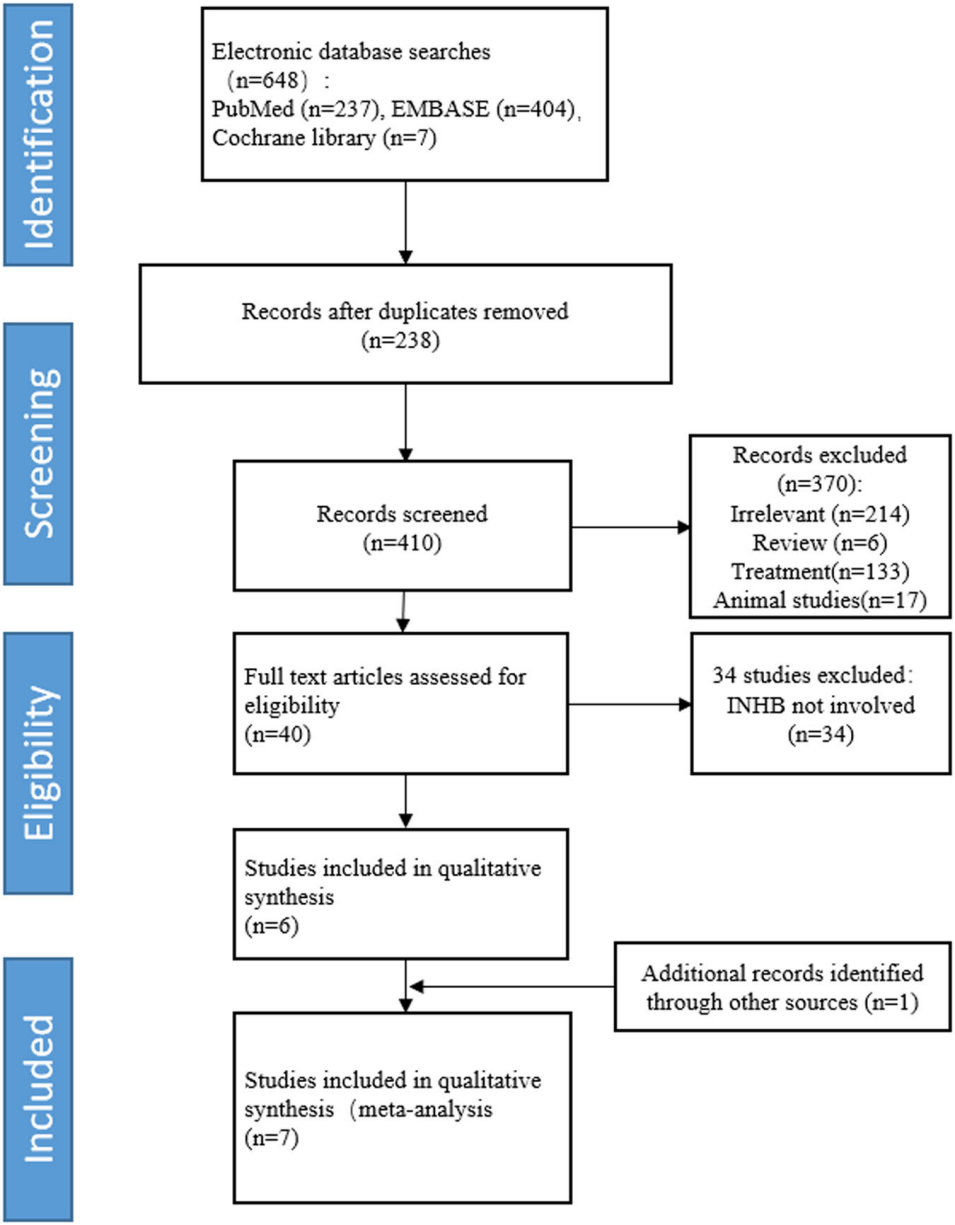
Flow diagram of study selection

A flow diagram of the literature search and study selection process is shown in Fig. 1. The initial database searches yielded 648 records. Of these, 238 were excluded after duplicates were removed, and 370 were excluded after screening the titles and abstracts. Thus, the full texts of 40 articles were reviewed. One article [24] was retrieved from the reference of included review. Finally, seven studies with 349 cases (96 HH and 253 CDGP) were included in the meta-analysis. The main reasons for exclusions were duplication of studies between PubMed and the EMBASE Databases, non-diagnostic research, and studies of INHB not relevant to the purposes of this study (Fig. 1).

### Study characteristics

Details of the included studies are summarized in Table 1. All were diagnostic studies published from 2010 to 2017, and provided sufficient data for the calculations required for this meta-analysis.

**Table 1:** The characteristics of included studies

Study ID	Coutant 2010 [28]			Binder 2015 [25]	Gerhard 2015 [40]	Rohayem 2015 [49]	Adan 2010 [24]	Sukumar 2017 [30]		Varimo 2017 [26]
	Coutant 2010 [28]^a^	Coutant 2010 [28]^b^	Coutant 2010 [28]^c^					Sukumar 2017 [30]^a^	Sukumar 2017 [30]^b^	
Classification methods										
TV (ml)	≤6	<3	3–6	≤4	Tanner breast stage B1/B2	<4	≤6	≤3		≤4
Priming Treatment of sex hormones	Unclear^a^			No	No	No	Unclear^a^	No	Injection of testosterone^b^	Unclear^a^
Inhibin B										
Cut-off value (pg/ml)	35	35	65	111	20	28.5	100	80	94.7	61
Measurement method	DSL ELISA			Gen II ELISA	Gen II ELISA	DSL ELISA	OBI ELISA	Gen II ELISA		OBI ELISA, Gen II ELISA
Standardized cut-off value^c^ (pg/ml)	66	66	113	111	20	56.0	96	80	94.7	61
True positive (*n*)	15	9	6	9	9	21	4	12	15	9
False positive (*n*)	0	0	2	4	0	6	13	1	0	10
False negative (*n*)	1	0	1	1	0	1	13	3	0	1
True negative (*n*)	51	23	26	48	12	18	26	14	15	50
Sensitivity rate	93%	100%	86%	100%	100%	95%	87%	80%	100%	90%
Specificity rate	100%	100%	92%	92%	100%	75%	90%	96%	100%	83%
Subject Number: CHH/CDGP (n)	16/51	9/23	7/28	9/52	9/12	22/24	15/39	15/15		10/60
Gender	Boys			Boys	Girls	Boys	Boys	Boys		Boys and girls
Age	14–18			13.7–17.5	13–17.5	13.9–23.2	14–17.4	14.1–26.2		14–18
Differential Diagnostic rules (“gold standard”)	Spontaneous and complete pubertal development			CHH:24 m of follow-up, TV < 5 ml CDGP:12–18 m follow-up, TV ≥ 8 ml;	Spontaneous complete pubertal development	Spontaneous complete pubertal development	Spontaneous complete pubertal development	Spontaneous and complete pubertal development		Spontaneous and complete pubertal development
TV measurement method	Unclear			Prader orchidometer	/	Unclear	Prader orchidometer	Ultrasound		Prader orchidometer
Basic information										
Study design	Prospective			Retrospective	Retrospective	Retrospective	Retrospective	Prospective		Retrospective
Town and country	Angers and Paris, France			Tuebingen, Germany	Tuebingen,Germany	Jeddah, Saudi Arabia	Paris, France	Chandigarh, India		Chandigarh, India
Journal	J Clin Endocrinol Metab			Clinical Endocrinology	The Journal of pediatrics	Andrology	Medical Science Monitor	Clinical Endocrinology		Human Reproduction

Coutant 2010 [28] a/b/c: The study was classified into three subset studies according to the subgroups of different testicular volume

Sukumar 2017 [30] a/b: The study was divided into two subset studies based on receiving a priming testosterone treatment or not

*CHH* congenital hypogonadotropic hypogonadism, *CDGP* constitutional delay of growth and puberty, *TV* testicular volume, *OBI* Oxford Bio-Innovation reagents, *DSL* Diagnostic Systems Laboratories, *ELISA* enzyme linked immunosorbent assay, *Gen II ELISA* second generation ELISA

^a^Prior sex hormone treatment history was not mentioned

^b^100 mg Testosterone intramuscular injection monthly for 3 months

^c^Assay method standardized to Gen II ELISA: Gen II = 1.03OBI − 6.77 pg/ml and Gen II = 1.57DSL + 11.29 pg/ml [27]

### Quality assessment

**Fig. 2:**
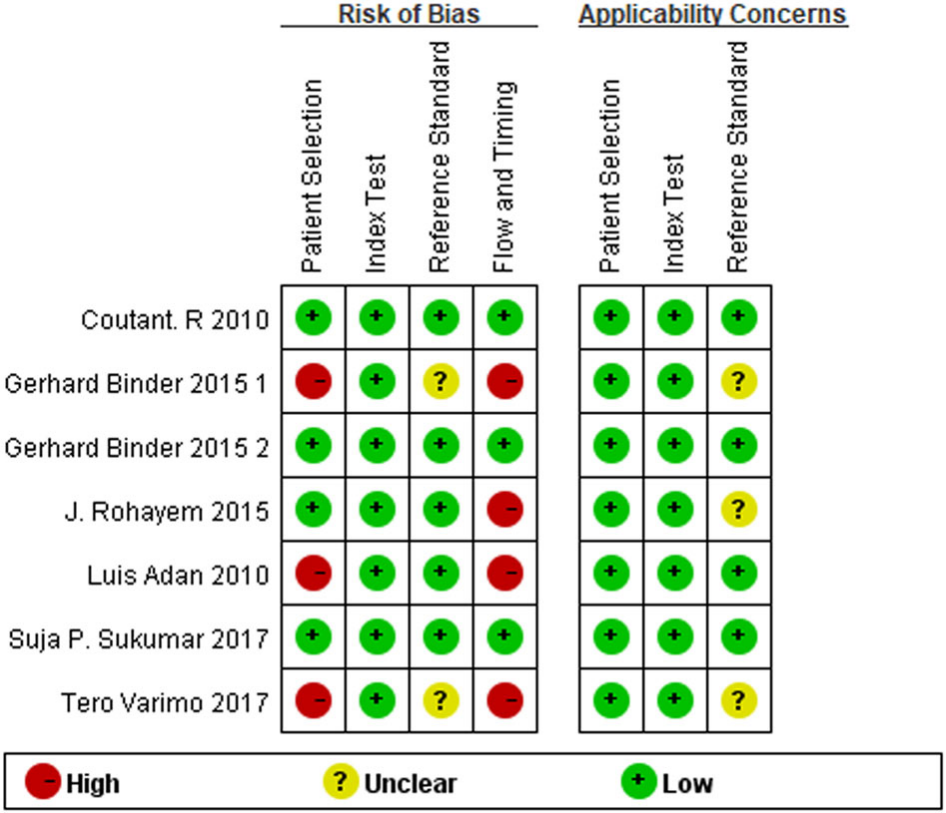
Quality assessment of the included studies using QUADAS-2 tool. Key: +, low risk; −, high risk; ?, unclear risk

The quality assessment of the included studies is summarized in Fig. 2. All studies fulfilled the criteria of the index test, i.e., the evaluation of INHB level was blind to the diagnosis. All studies except one used spontaneous complete pubertal development as the reference diagnostic standard. In the study by Binder et al. [25] six boys had a TV between 5 and 8 ml at 24 months of follow-up, which did not fulfill the definition of either CHH or CDGP defined in their study and were therefore considered inappropriate exclusions. In terms of patient selection, all seven studies had a low risk of bias, and mentioned whether they included consecutive patients. However, three of the studies excluded patients for unclear or unreasonable standards [24–26]. With respect to flow and timing bias, other than the abovementioned three studies, one study [24] ignored a CHH patient (16 CHH patients were included in the table, but only 15 were analyzed) for an unclear reason, which was considered a potential source of bias. Overall, none of the seven studies were excluded from this meta-analysis due to methodological flaws.

### Accuracy of INHB for distinguishing CHH and CDGP

**Fig. 3:**
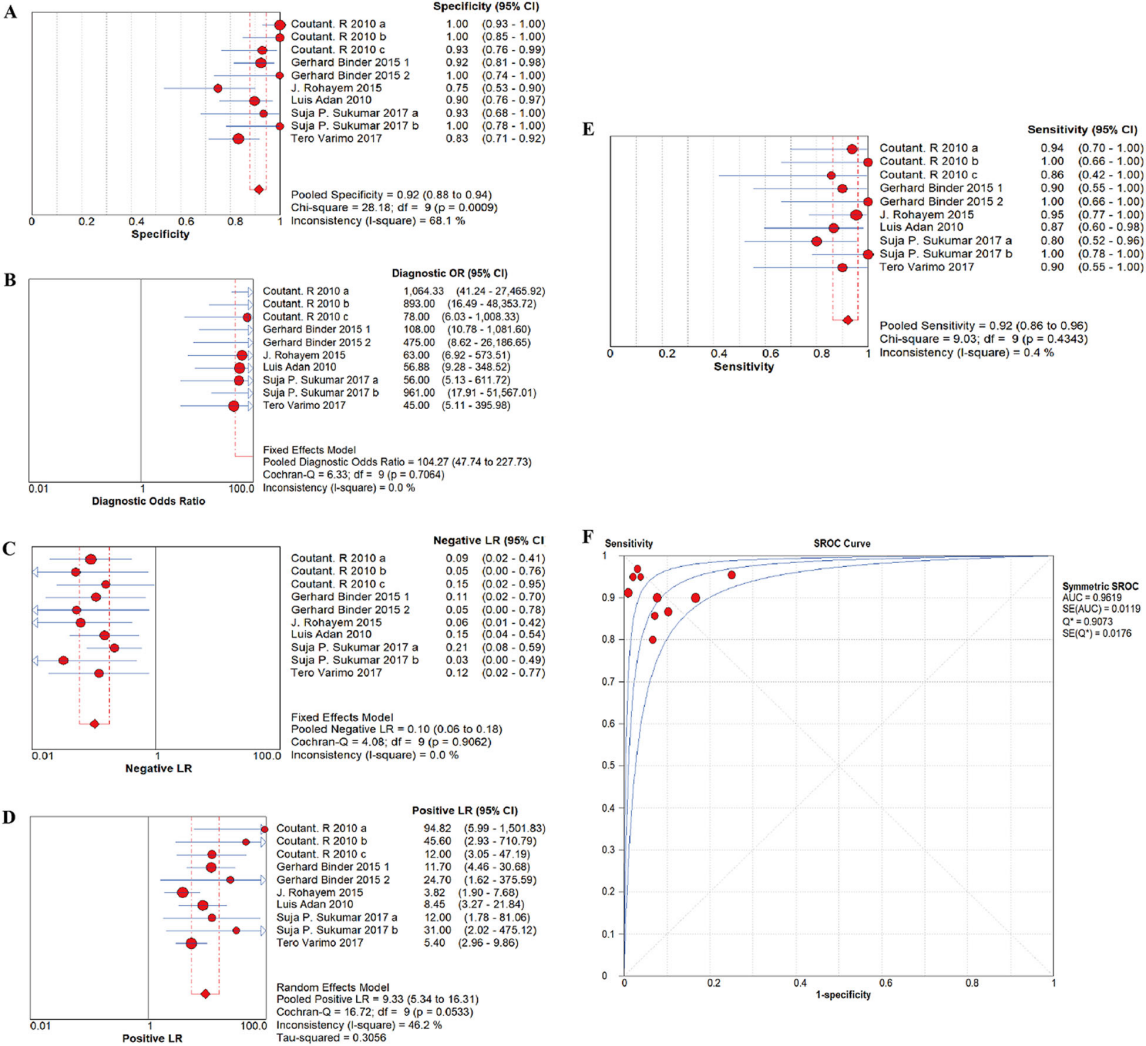
Forest plot for sensitivity (**a**), specificity (**b**), Positive likelihood ratio (LR) (**c**), Negative LR (**d**), pooled diagnostic odds ratio (DOR) (**e**), of eligible studies for INHB in diagnosis. The results are represented with study estimate, summary receiver-operating characteristic (sROC) curves and 95% confidence region (**f**)

The diagnostic performance results for serum INHB level in differentiating CHH from CDGP are shown in Fig. 3. The pooled sensitivity and specificity (random-effect model) for serum INHB in distinguishing the two conditions were 0.92 (95% CI: 0.86–0.96) and 0.92 (95% CI: 0.88–0.94), respectively. There was a considerable level of heterogeneity for the sensitivity (*I*^2^ = 0.4%, *p* = 0.4343) and for specificity (*I*^2^ = 68.1%, *p* = 0.0009). The pooled DOR was 104.27 (95% CI: 47.74–227.73), with low heterogeneity (*I*^2^ = 0.00%, *p* = 0.6158). The pooled positive likelihood ratio (PLR) and negative likelihood ratio (NLR) were 9.33 (95% CI: 5.34–16.31, *I*^2^ = 46.2%, *p* = 0.0533) and 0.10 (95% CI: 0.06–0.18, *I*^2^ = 0.0%, *p* = 0.9062), respectively. The AUC was 0.9619, and the Q* was 0.9073. The Spearman correlation coefficient was −0.417 (*p* = 0.3082), suggesting no threshold effect existed.

### Exploration of heterogeneity

In the pooled analysis, the included studies were statistically heterogeneous in their estimate of specificity. Potential heterogeneity in sensitivity and specificity was explored with subgroup analysis. TV, study design, methodological quality, publication year, and gender were used as co-variants in subgroup analysis. The results suggested that TV and study design could influence the diagnostic value. It is worth noting that subgroup analysis by TV revealed that the pooled sensitivity, specificity, and AUC in CHH subjects with profound gonadotropin deficiency (as indicated by TV ≤ 3 ml) were 0.92, 0.98, and 0.9956, respectively, which exhibited the best diagnostic efficacy with low heterogeneity of specificity. The pooled sensitivity and specificity of prospective studies were 0.93 and 0.99, respectively, with lower heterogeneity. The pooled sensitivity, specificity, and AUC for serum INHB in the boys with delayed puberty were 0.92, 0.93, and 0.9645, respectively. Details of subgroup analyses are shown in Table 2.

**Table 2:** Summary results of subgroup analysis for INHB in the diagnosis between CHH and CDGP

Categorical variable	No. of	Sensitivity			Specificity		
	studies	95% CI	*I*^2^(%)	*P* value	95% CI	*I*^2^(%)	*P* value
Testicular volume(ml)							
≤3	3	0.92(0.79–0.98)	67.4	0.0464	0.98(0.90–1.00)	22.3	0.2761
≤4	3	0.93(0.81–0.99)	0.00	0.7884	0.85(0.78–0.91)	53.7	0.1155
≤6	2	0.90(0.74–0.98)	0.00	0.5021	0.96(0.89–0.99)	85.6	0.0085
Study design							
Prospective	4	0.93(0.82–0.98)	51.4	0.1033	0.99(0.95–1.00)	23.7	0.2690
Retrospective	5	0.92(0.83–0.97)	0.00	0.6457	0.87(0.82–0.92)	51.6	0.0822
Methodological quality							
Low risk	5	0.94(0.85–0.98)	46.2	0.1147	0.99(0.95–1.00)	3.6	0.3860
Not low risk	4	0.91(0.81–0.97)	0.00	0.8098	0.86(0.80–0.91)	38.2	0.1828
Publication year							
2010	3	0.93(0.80–0.98)	2.40	0.3589	0.96(0.91–0.99)	77.3	0.0123
2015	3	0.95(0.83–0.99)	0.00	0.5104	0.89(0.80–0.94)	71.9	0.0285
2017	3	0.90(0.76–0.97)	55.5	0.1058	0.88(0.79–0.94)	63.1	0.0664
Gender							
Boys only	7	0.92(0.85–0.97)	16.4	0.3052	0.93(0.89–0.96)	71.5	0.0018
Girls included	2	0.95(0.74–1.00)	25.0	0.2482	0.86(0.76–0.93)	74.7	0.0467

### Publication bias

Since CHH is a rare disease, the number of studies in each method group was small (<10), and it was difficult to draw funnel plots to examine publication bias. An exhaustive search was performed in the PubMed, EMBASE, and Cochrane Controlled Register databases, and references of related issues were examined to minimize publication bias.

## Discussion

During early adolescence, distinguishing CHH and CDGP is extremely challenging, as a delay in puberty is a hallmark of both diseases and both have HH as a characteristic. Whereas GnRH deficiency is permanent in most cases of CHH, CDGP is a state of transient GnRH deficiency where puberty eventually begins and is completed without hormonal treatment [1]. In addition, CDGP is a common cause of delayed puberty, whereas CHH is considerably rarer. Differentiating CHH from CDGP is crucial to avoid postponing hormonal therapy and alleviate the psychological burden associated with delayed sexual maturation [5]. In addition, differentiating a transient condition from a chronic disease can influence a patient’s quality of life [5]. This meta-analysis indicated that serum INHB level has good diagnostic accuracy for differentiating the two conditions, with a pooled sensitivity of 92%, specificity of 92%, and a pooled AUC of 0.9619. It also performed especially well in boys with severe puberty deficiency (TV ≤ 3 ml) with a sensitivity of 92%, specificity of 98%, and AUC of 0.9956. The INHB DOR was 104.27, suggesting it is a useful method to identify CHH. A PLR of 9.33 determined in this meta-analysis indicated that patients with an INHB level lower than the cut-off value were nine times more likely to have CHH. In contrast, an NLR of 0.10 suggested that an IHNB level higher than the cut-off value was associated with a 90% chance of having CDGP.

### Variation in the cut-off value of INHB

In this study, we examined seven cut-off values of INHB level in boys with DP (28.5 pg/ml, 35 pg/ml, 65 pg/ml, 80 pg/ml, 94.7 pg/ml, 100 pg/ml, and 111 pg/ml). Each of the cut-off values exhibited good diagnostic accuracy. The best diagnostic accuracies were observed in the case of severe puberty deficiency (TV ≤ 3 ml) with the cut-off value of 35 pg/ml, and in the situation of discontinuing “3-month testosterone priming” with a cut-off value of 94.7 pg/ml; in both instances the sensitivity and specificity were 100%. There are several reasons for choosing the various cut-off values.

The first reason is the variation between INHB measurement methods, instruments, and lab techniques. In order to minimize the variation, the Oxford Bio-Innovation reagents (OBI) assay and Diagnostic Systems Laboratories (DSL) assay were standardized to the second-generation enzyme-linked immunosorbent assay (Gen II ELISA). The conversion formulas used were Gen II = 1.03 × OBI − 6.77 pg/ml; Gen II = 1.57 × DSL + 11.29 pg/ml [27]. Hence, the converted cut-off values are 56 pg/ml, 66 pg/ml, 80 pg/ml, 96 pg/ml, 94.7 pg/ml, 111 pg/ml, and 113 pg/ml, which narrowed the range of INHB for differentiating CHH and CDGP (Table 1).

Secondly, different baseline TV greatly impacted the diagnostic cut-off value of INHB. The results of subgroup analysis suggested that heterogeneity mainly stemmed from the TV value, and also suggested that TV might influence the cut-off value. In the study by Coutant et al. [28], the cut-off value in boys was 35 pg/ml at genital stage 1, and 65 pg/ml at stage 2. INHB is secreted by Sertoli cells and reflects Sertoli cell number and function, which correlates well with testicular size. A TV ≤ 3 ml indicates that the patient has profound gonadotropin deficiency and a relatively lower serum INHB level. Another important reason for using different cut-off values is the TV assessment method. The measurement of TV can vary markedly between different examiners and measurement methods. It is usually suggested that there are only a few designated examiners to perform TV assessment with a Prader orchidometer. In our experience, the TV measured with a Prader orchidometer is overestimated by 4.5 ± 2.7 ml (3.2 times) compared to the ultrasonographic calculation of TV in CHH [29].

Thirdly, the cut-off value is affected by different sampling conditions; a naïve assessment without any intervention treatment, or after testosterone treatment. Sukumar et al. [30] demonstrated that an INHB level >94.7 pg/ml was discriminatory for diagnosing CDGP after withdrawal of testosterone priming, with a sensitivity of 100% and specificity of 100%. On the other hand, basal INHB levels >80 ng/ml prior to testosterone therapy had a sensitivity of 80% and specificity of 96%. Thus, prior treatment with sex hormones might influence the cut-off value of INHB to some extent. Prior treatment with sex hormones might vary in these studies.

Lastly, CHH is a very heterogeneous disorder and includes reversal of CHH [31, 32], partial CHH [33], and CHH occurring in adult patients after normal pubertal development [34], which makes its diagnosis complicated. CHH patients might have normal adult testosterone levels after discontinuing hormonal therapy, otherwise known as reversal of CHH. Reversibility occurs in both males and females with CHH, and it is more common (10– 20% in males, and a few case reports for females) than previously thought [32]. Partial CHH typically presents with mild gonadotropin deficiency and partial puberty development [33].

The cut-off value for INHB in boys ranged from 56 to 113 pg/ml (Gen II ELISA), suggesting a variance resulting from the patient’s condition and hospital laboratory testing variances. Although this study could not confirm a single, optimal cut-off value, the results do indicate that IHNB is an excellent diagnostic marker in differentiating CHH from CDGP, and that every center should determine its own cut-off values of INHB.

### INHB in girls with CHH or CDGP

In contrast to boys with DP, there is a paucity of studies of girls with DP [35]. Most studies included in this meta-analysis were focused on prepubertal boys. The prevalence of girls with CDGP is approximately five times lower than that of boys [36]. Only one randomized study included several girls with CHH [37]. Several articles have reported a lower INHB concentration in prepubertal girls [38, 39]. Binder et al. [40] demonstrated a basal INHB cut-off of 20 pg/ml (Gen II ELISA). Similar results were reported by Juul et al. [41], which reported a lower reference range of 20 pg/ml for INHB for healthy girls at Tanner stage B2.

### Recent studies examining the discrimination of CHH from CDGP

We reviewed some features that may assist in the differential diagnosis, noting that although individual indicators may not provide a definitive resolution, a combination of multiple indicators and clinical observations can strengthen arguments for or against a particular diagnosis. New investigations regarding parameters for distinguishing CHH and CDGP are summarized below.

Some clinical features can potentially distinguish CDGP from CHH, although they do not have high diagnostic value. The presence of cryptorchidism and/or micropenis favors a diagnosis of CHH, and indicates the absence of gonadotropins and sexual hormones during both fetal life and minipuberty [1, 30]. Congenital hyposmia or anosmia are not consistent with a diagnosis of CDGP [42]. TV is useful for discriminating CHH from CDGP in boys. In a study of 174 boys with DP [30], those with a TV < 1.1 ml (measured by Prader orchidometer) showed a 100% sensitivity and 91% specificity in distinguishing CHH from CDGP. Growth velocity has been shown to offer no additional diagnostic value in distinguishing between CDGP and CHH [43]. Family history is also not helpful for distinguishing the two conditions because individuals with CHH often have family members with CDGP [44].

Genetic testing shows promise for distinguishing the two conditions, though more studies are needed. Cassatella et al. [45] suggested that CDGP and CHH have distinct genetic profiles; CHH has gene mutations in 51% of the probands, but CDGP has mutations in only 7% of probands.

It is challenging to make a clinical distinction between CHH and CDGP based on other biochemical markers because most biochemical markers are not always discriminatory. To date, there is no biochemical marker that can fully differentiate CHH from CDGP in early adolescences. Harrington and Palmert [46] systematically reviewed studies of basal gonadotropin levels (e.g., LH, FSH, testosterone), and reported that gonadotropin levels had limited ability in distinguishing between CHH and CDGP, primarily because of the overlap of gonadotropin levels. There had been many attempts to use stimulation tests, including GnRH and GnRHa stimulation tests, and HCG testing, to distinguish between the two conditions. A GnRH test has been considered useful for identifying CHH; CHH is highly probable when a GnRH-stimulated LH response is blunted. Varimo et al. [26] demonstrated a peak LH cut-off value of 4.3 IU/L post GnRH stimulation test had a sensitivity of 100% and specificity of 75% for detecting CHH. Another study [47] showed that a peak LH < 9.74 IU/L had moderate sensitivity (80.0%) and specificity (86.4%) for diagnosing CHH in males. However, different stimulation methods with GnRH or GnRHa, and different dosages of GnRHa have resulted in a variety of peak LH cut-off values [9, 48]. In HCG stimulation tests, different HCG dosages, injection frequency, and timing of blood sampling affect the results of testosterone measurements [11–13]. In a study of 14 CHH and 29 CDGP patients [46], HCG 1500 IU was prescribed on days 1, 3, and 4 and testosterone level was measured on days 1 and 5. A testosterone level of <1.04 µg/L on day 5 showed a 92% sensitivity and 92% specificity for diagnosis. When HCG 1500 IU was injected on days 1, 3, 4, 9, 12, 16, and 19 and testosterone was measured on days 1, 5, and 20 a testosterone level of <2.75 µg/L on day 20 exhibited a 92% sensitivity and 95% specificity for distinguishing CHH from CDGP. All of the aforementioned results are inferior to that of using a baseline INHB level, which was found to have 93% sensitivity and 100% specificity in this meta-analysis.

The frequent injections and samplings of stimulation tests make them impractical for routine use. Some studies have examined the diagnostic utility of anti-Mȕllerian hormone (AMH), which is secreted by Sertoli cells. A lower AMH level is suggestive of CHH, but it is less accurate than INHB. However, AMH had distinctive efficacy for pre-pubertal boys with a bilateral TV ≤ 8 ml. AMH levels are significantly higher in CDGP boys than CHH boys during pre-puberty [49]. Normally, AMH levels do not change in a linear manner over the pubertal transition period, but rather fluctuate [28, 30]. AMH declines transiently after birth, then increases to a peak at 2–4 years old and remains elevated during pre-puberty. Afterward, it decreases sharply with puberty development. There was an overlap of AMH levels between subgroups, which is likely because of the lower diagnostic efficacy of AMH values relative to INHB in distinguishing CDGP and CHH. Coutant [28] described a cut-off value of 110 pmol/l (15.4 ng/ml) analyzed by electrochemiluminescence immunoassay (ECLIA). Rohayem et al. [49] showed a cut-off value of 20 ng/ml using an ELISA could differentiate between CDGP and CHH. Other markers such as insulin-like factor (INSL), dehydroepiandrosterone sulfate, and insulin-like growth factor (IGF)-1 did not improve the accuracy of diagnosis [50].

Coutant et al. [28] found that the combination of INHB and LH (with cut-offs of 35 pg/ml and 0.5 mIU/L, respectively) offered 100% sensitivity and specificity in differentiating between CHH and CDGP in adolescents with a TV ≤ 3 ml. According to our meta-analysis results, and a comparison of the results with the above-mentioned approaches, a single measurement of INHB level provides good diagnostic accuracy and is far less costly.

### Strengths and limitations

This was the first meta-analysis that comprehensively explored the diagnostic efficiency of INHB in distinguishing CHH from CDGP, and provided a quantitative analysis. However, this study had some limitations. The number of studies and patients was relatively low because CHH is a rare disease, and some studies had low methodological quality. We could not determine a single, optimal cut-off value. Less than ten studies were included in the meta-analysis, and thus the results of the subgroup analyses must be treated with caution. Lastly, potential publication bias might exist because only studies published in English were included.

## Conclusion

INHB level is useful for differentiation between CHH and CDGP, especially in boys with PD and a TV ≤ 3 ml. An INHB reference range of 56–113 pg/ml for boys and 20 pg/ml for girls (Gen II ELISA) may provide useful information for discrimination between CHH and CDGP. Additional studies with large sample sizes and standardized methodology would be required to achieve a more robust and credible result. A combination of multiple parameters, such as clinical characteristics, laboratory data, and genetic sequencing may ultimately provide high diagnostic accuracy and determination of prognosis.
